# *In-vitro* analysis of free radical scavenging activities and suppression of LPS-induced ROS production in macrophage cells by *Solanum sisymbriifolium* extracts

**DOI:** 10.1038/s41598-020-63491-w

**Published:** 2020-04-16

**Authors:** Garland K. More, Raymond T. Makola

**Affiliations:** 1 College of Agriculture and Environmental Sciences, CAES Laboratories, University of South Africa, Private Bag X6, Florida Johannesburg, 1710 South Africa; 2 Department of Biochemistry Microbiology and Biotechnology, School of Molecular and Life Science, University of Limpopo (Turfloop Campus), Sovenga, 0727 South Africa; 3 National Institute for Communicable Diseases, Special Viral Pathogen/Arbovirus Unit, 1 Modderfontein Rd, Sandringham, Johannesburg 2192 South Africa

**Keywords:** Bacterial infection, Inflammasome

## Abstract

The current study aims to evaluate the antioxidant, cytotoxicity activities and suppression of LPS-induced oxidative stress production and characterization of phytochemicals in *Solanum sisymbriifolium* leaf extracts. The 2,2-diphenyl-1-picrylhydrazyl (DPPH) and 2,2′-azino-bis3-ethylbenzothiazoline-6-sulphonic acid (ABTS) radical scavenging activity of the leaves of *S. sisymbriifolium* extracted with solvents of various polarities viz. water: ethanol, ratio 50: 50; ethyl acetate and dichloromethane, was assessed. The cytotoxicity of the extracts was determined using the [3-(4, 5-dimethylthiazol-2-yl)-2, 5-diphenyltetrazolium bromide] (MTT) assay on RAW 264.7 macrophage (Murine) cells and real-time cell analysis (RTCA) xCELLigence system was used for determining cell viability. Cell-based detection of reactive oxygen species (ROS) was investigated utilizing a 2′,7′-Dichlorodihydrofluorescein diacetate (H_2_DCF-DA) assay. The DPPH and ABTS scavenging activity results of extracts revealed a dose-dependent response with significantly lower activity in both DPPH and ABTS. The superoxide dismutase (SOD) enzyme activity was then evaluated and extracts displayed a high SOD enzyme activity with 90–50% activity. Cytotoxicity results revealed that *S. sisymbriifolium* extracts were not toxic to RAW 264.7 macrophage cells at the tested concentrations. All three extracts decreased the production of ROS in macrophage cells. Phytochemical analysis using Fourier-transform infrared spectroscopy (FTIR) indicated the presence of metabolite functional groups which may be responsible for the antioxidant activity. The current study indicates that *S. sisymbriifolium* contains phytochemicals that scavenge free radicals, with less toxicity, and suppresses the LPS-induced ROS production in RAW 264.7 macrophage cells.

## Introduction

The escalation in oxidative stress-related diseases has become a major concern worldwide. Cancer, diabetes, cardiovascular disorders, neurodegenerative diseases, inflammation, cataracts, and osteoporosis are some of the diseases which directly or indirectly originate from excessive production of reactive oxygen/nitrogen species (ROS/RNS) in living cells^1^. Oxidative stress is a condition where oxidants overwhelm the antioxidant protective system, which may lead to DNA damage and cellular lipid peroxidation. An imbalance between the production of antioxidants such as superoxide dismutase, glutathione peroxidase, and catalase as well as the non-enzymatic antioxidants such as glutathione, vitamin C, E and D, and the production of free radicals (reactive oxygen and nitrogen species) elicits an oxidative stress condition. Superoxide (O_2_^−^) is the primary ROS, generated through the reduction of oxygen (O_2_) molecules by NADPH oxidase during mitochondrial respiratory electron transport chain. This process leads to an accumulation of ROS like hydrogen peroxide (H_2_O_2_), generated by an enzyme superoxide dismutase^2^. Throughout oxidative stress, excess H_2_O_2_ forms toxic ROS like hydroxyl ions (HO^−^) through a catalytic process in the presence of reduced metals (Cu, Fe and Ni)^2^. During these conditions, reactive molecules have been shown to target proteins, lipids and nucleic acids resulting in an alteration of cell structures and functions^3^. Due to low glutathione content and high proportions of polyunsaturated fatty acids in membranes in neuronal cells, lipid peroxidation has been observed in patients suffering from Alzheimer’s disease (AD), Parkinson’s disease (PD) and stroke. Hydroxyl ions are the most reactive species that damage DNA through the formation of 8-hydroxydeoxyguanosine which is known to be a radical attack biomarker^2^. Oxidative modification of low-density lipoprotein (LDL) in the arterial walls is thought to cause endothelial dysfunction which is the first stage of atherosclerosis^4^. These increased levels of ROS can be reduced to normal levels by enhancing cellular antioxidants that promote protective reactions in the cells. Conversely, low levels of ROS play a beneficial role in cell signaling pathways that stimulate cell survival^5^. Cells in the presence of reduced ROS induce cellular enzymes such as superoxide dismutase (SOD), catalase (CAT) and glutathione peroxidase (GSHPx) that play a fundamental role in modulating free radicals into H_2_O_2_ and converting them into H_2_O^2,3^. Studies by Morgan and Liu^6^; Barnes and Karin^7^ and Baker^8^ have outlined the link between oxidative stress and chronic ailments with prolonged activation of activators such as protein-1 (AP-1), nuclear factor (NF-κB) and glycogen synthase kinase-3 beta (GSK-3β), which are the vital inflammatory mediators. Macrophage cells, which serve as the first line of defense in infected cells, act to suppress the multiplication and spread of pathogens by producing inflammatory mediators and by phagocytosing pathogens. During the invasion of cells by pathogens, pathogen-associated molecules such as lipopolysaccharides (LPS), initiates an inflammatory response. Through this process, cytokines including tumour necrosis (TNF-α), interleukin (IL-1β) and prostaglandin (PGE_2_) play a vital role in normalizing ROS levels. However, phytochemicals such as vitamins, polyphenols, carotenoids and flavonoids are known as scavengers of free radicals^9^. These natural antioxidants quench peroxyl and/or hydroxyl radicals by donating an electron to them and in the process become free radicals themselves, but of lesser damaging effects.

Medicinal plants, which are the basis of traditional medicine in various countries, have been investigated for their pharmacological properties and have revealed numerous significant novel phytochemicals with the potential to treat and manage diseases^10^. This includes the discovery of paclitaxel (Taxol), an anticancer drug discovered from the bark of the Himalayan Yew tree^11^; berberine isoquinoline alkaloid an antineoplastic, antidiabetic drug found in plants belonging to Berberidaceae family^12^, and Artemisinin, an antimalaria drug derived from *Artemisia annua*^13^, to mention a few. In recent studies, plants and their constituents have shown significant potential as therapeutic agents for oxidative stress and include polyphenolic compounds such as resveratrol, gallic acid, catechin, quercetin, kaempferol, and several anthocyanins from grapes skin^14^. A review documented by Mattioli^15^, presents the importance of plant-derived bioactive phytochemicals that possess activity against oxidative stress in chronic, degenerative, and infectious diseases. These include catalpol from the roots of *Rehmannia glutinosa* which was found to inhibit ROS production, DNA damage, and telomere shortening. However, the review further highlights the antitumor effects of *the Annona muricate* plant and the antioxidant, antidiabetic, and anti-obesity effects of *Citrus aurantium*. Despite efforts to discover new therapeutic agents, oxidative stress-related diseases such as cancer, neurodegenerative disorders, cardiovascular and metabolic diseases still form part of the highest causes of death worldwide which warrants further research to be conducted with a new approach to fight these diseases.

*Solanum sisymbriifolium* Lam. (Solanaceae) commonly known as “wild tomato” is a plant that is native to Central America and is found in the Chaco Biome of South Paraguay. It is considered a highly invasive weed in South Africa and is widely distributed in the Eastern and Western Cape, KwaZulu-Natal, Mpumalanga, Gauteng and Limpopo Province. Moreover, a review has documented the medicinal uses of *S. sisymbriifolium* by the native people of South America to treat a wide range of ailments including febrifuge, syphilis, hypertension, diarrhoea, urinary tract infections. It is also used as a precursor for contraceptives and a hepatoprotective remedy^16^. Studies have demonstrated the pharmacological properties of *S. sisymbriifolium* including antimicrobial^17^, anti-fungal^18^, antioxidant^17^, molluscicidal, piscicidal and insecticidal^4,19^, anti-inflammatory, analgesic, anti-diarrhoeal and anti-diabetic^20^ activities. Furthermore, other studies have highlighted the anti-hypertensive and cardio-protective effects exhibited by a different part of *S. sisymbriifolium*^10,11^. Solasodine, a compound commonly found in the *Solanaceae* family has previously been isolated from *S*. *sisymbriifolium* and exhibits protective properties against ischaemic stroke in rat brains^15^. The mechanism of this mode of protection was via the antioxidant system which enabled the stimulation of superoxide dismutase (SOD), catalase (CAT) and glutathione (GSH), which ultimately led to a reduction in lipid peroxidation (LPO) and nitric oxide (NO) levels in these rats^21^. However, literature is limited regarding the antioxidant activities, especially the modulation of reactive oxygen/nitrogen species (ROS) and the cytotoxicity on macrophage cells by extracts of *S. sisymbriifolium*.

The main aim of this study was to investigate the antioxidant activity of *S. sisymbriifolium* leaf extracts utilizing DPPH and ABTS radical scavenging photometric assays and determine the LPS-induced ROS in RAW 264.7 macrophage cells *in-vitro*. DPPH is an organic compound that is composed of stable free radical molecules and presents as a dark purple powder which turns clear when reduced by an antioxidant. ABTS cation is a deep blue-green solution in the presence of potassium per-sulfate which turns light-green when reduced by hydrogen-donating antioxidants^22^. The LPS-induced ROS in RAW 264.7 macrophage cells was performed using the H_2_DCF-DA fluorescent dye assay. This assay is based on the principles of the reaction of H_2_DCF with ROS to form a fluorescent DCF^23^, where the intensity of the fluorescence embodies the levels of ROS produced. These reactions are then quantified by measuring the fluorescence spectrophotometrically.

## Results

### DPPH scavenging activity

**Figure 1:**
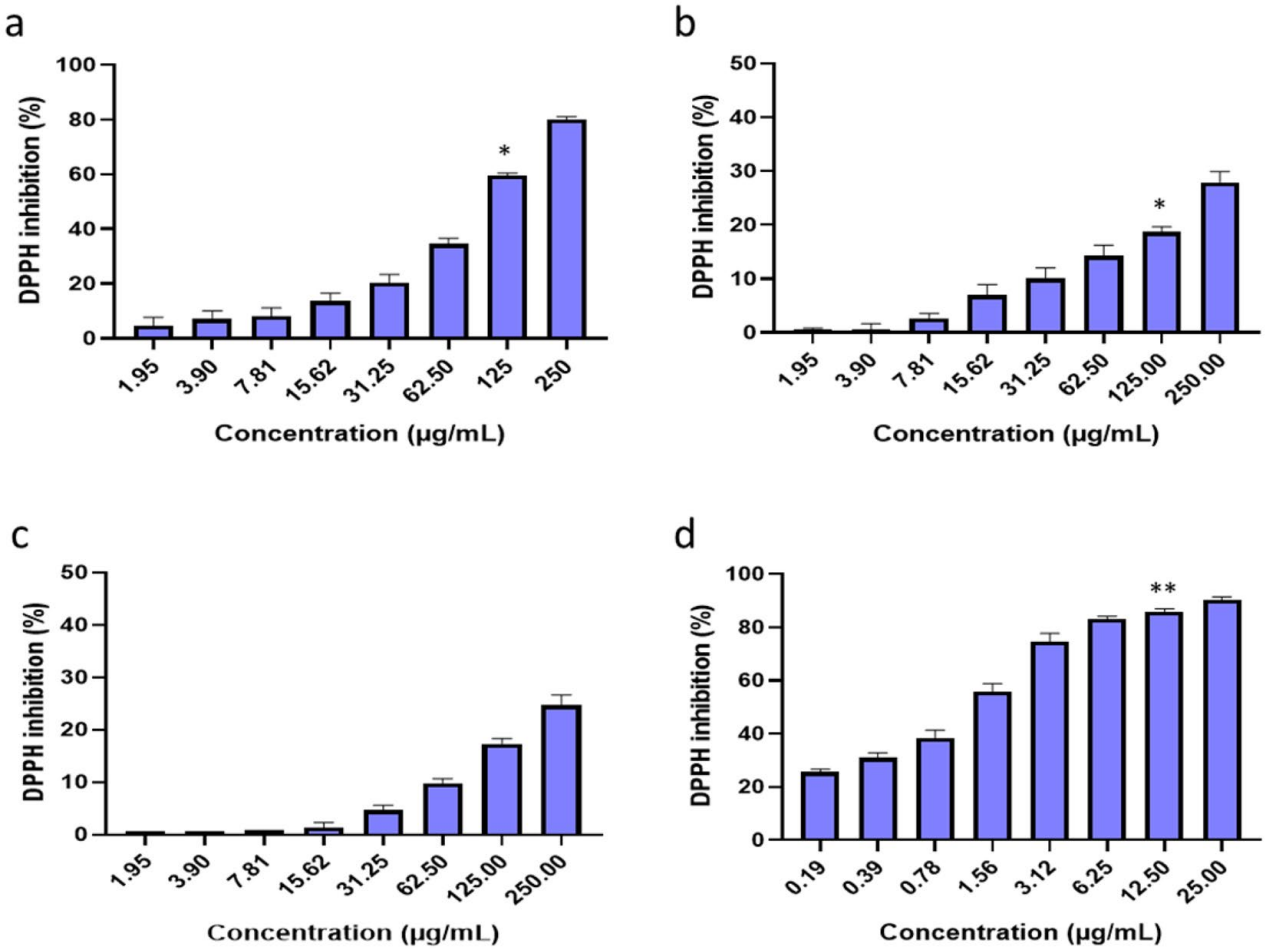
DPPH radical scavenging activities of various concentrations of *S. sisymbriifolium* leaf phytochemicals extracted with different solvents including 50% ethanol **(a)**, ethyl-acetate **(b)**, dichloromethane **(c)** and positive control (Ascorbic acid) **(d)** and the goodness of fit of R^2^ ≥ 0. 900. The bar graphs with asterisks (*), (**) denotes a significant difference (p ≤ 0.05), (p ≤ 0.01) when compared to the control based on Duncan’s multiple comparison test. Data is represented as Mean ± SD, n = 3.

*In-vitro* scavenging activity of *S. sisymbriifolium* extracts using non-enzymatic DPPH, ABTS radicals, enzymatic SOD inhibitory activity and cell-based LPS-induced ROS production on macrophage cells were evaluated. The radical scavenging activity of *S. sisymbriifolium* 50% ethanol, ethyl-acetate and dichloromethane (WE, EAA and DCM) extracts was determined using the DPPH scavenging assay. The radical scavenging activity of the extracts showed that the WE extract inhibited 80% - 20% at 250–31.25 µg/mL, with an EC_50_ value of 131.1 µg/mL (Fig. 1a). The EAA and DCM extracts had the lowest inhibitory activity with approximately 30% and 25% scavenging activity at the highest concentration tested, respectively (Fig. 1b,c). The EC_50_ for the EAA and DCM extract was 426.3 and 474.0 µg/mL, respectively. Results obtained from these extracts were higher than the positive control (ascorbic acid), which displayed 80–90% DPPH scavenging activity at 10x less concentration range of 25 to 0.3906 µg/mL with an EC_50_ of 4.0 µg/mL (Fig. 1d). The results obtained in this study are similar to the study conducted by Gupta *et al*.^17^, where an *S. sisymbriifolium* methanol extract tested at different concentrations (300, 200, 100, 80, 60 and 40 µg/mL) showed a low DPPH radical scavenging activity with an EC_50_ of 211.4 µg/mL, as compared to the positive control Quercetin, with an EC_50_ of 1.85 µg/mL. Similarly, the results from the controls of the fruit ethanol extract of *S. sisymbriifolium* against DPPH radical showed EC_50_ values of 21 and 10 µg/mL at concentrations of 100 and 50 µg/mL where the required inhibition was ≥50% at 50 µg/mL^24^.

### ABTS scavenging activity

**Figure 2:**
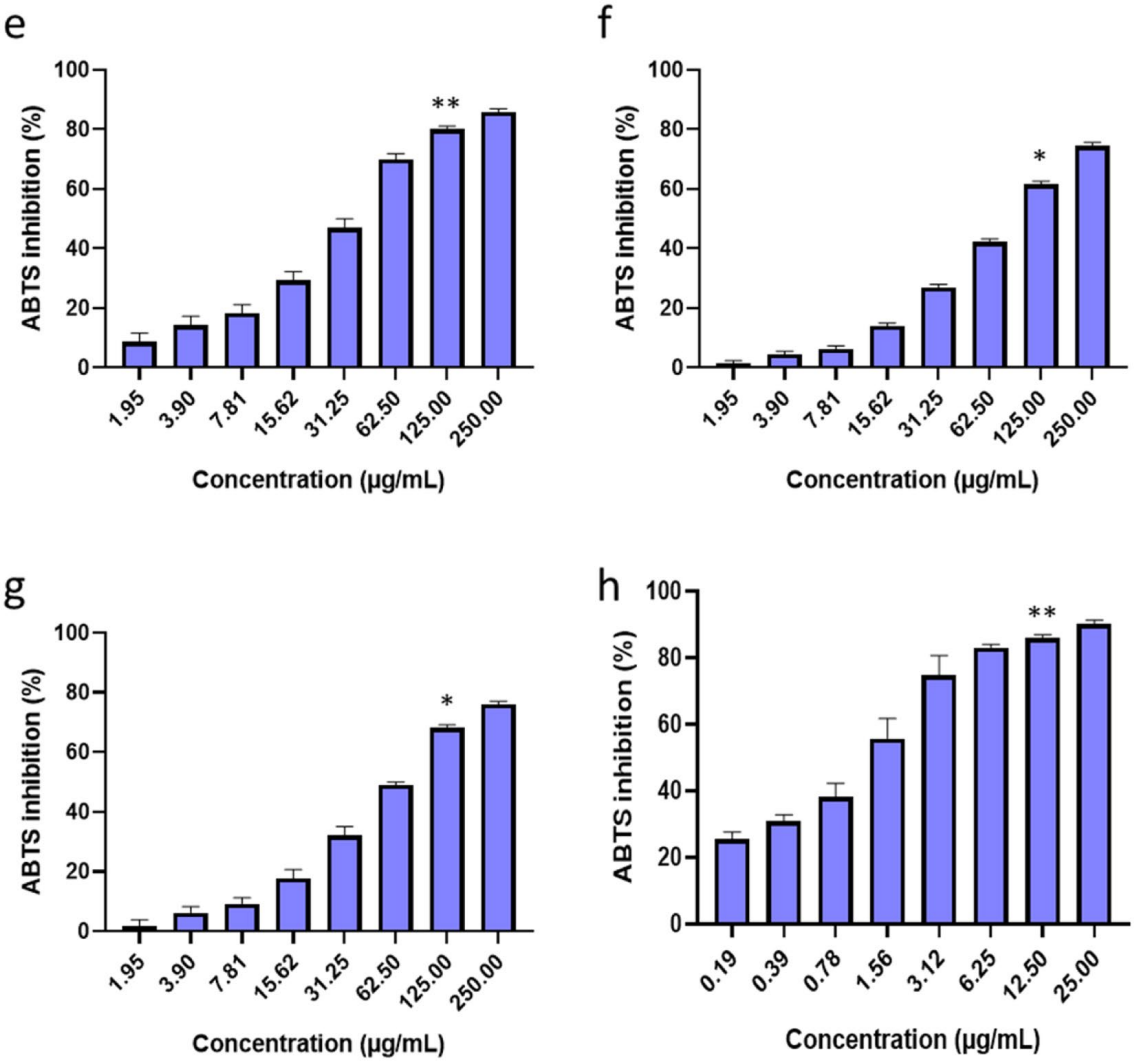
ABTS cation radical scavenging activities of various concentrations of *S. sisymbriifolium* leaf phytochemicals extracted with different solvents including those of 50% ethanol **(e)**, ethyl-acetate **(f)**, dichloromethane **(g)** and ascorbic acid **(h)** and the goodness of fit of R^2^ ≥ 0. 900. The bar graphs with asterisks (*), (**) denotes a significant difference (p ≤ 0.05), (p ≤ 0.01) when compared to the control based on Duncan’s multiple comparison test. Data is represented as Mean ± SD, n = 3.

The scavenging activity of *S. sisymbriifolium* leaf extracts tested at the concentration range of 250 to 1. 953 μg/mL was assessed using the ABTS cation assay. The EAA and DCM extracts were the least active extracts with an inhibitory activity ≤70% at the highest concentration tested, while the WE extract showed inhibitory percentage activity ≥70% (Fig. 2e). The WE extract exhibited an EC_50_ of 39.4 μg/mL, followed by the DCM and EAA extracts, which had an EC_50_ of 60.5 and 92.0 μg/mL, respectively. Ascorbic acid served as a positive control and was tested at 10x lower concentrations (25 to 0.195 μg/mL) to that of the extracts. The ascorbic acid exhibited inhibitory activity against the ABTS cation of 50–90% at concentration ranges of 25–1.563 μg/mL (Fig. 2h) and the EC_50_ was calculated to be 1.50 μg/mL. The ABTS cation scavenging activity results of the extracts and the positive control were not significant as they had large EC_50_ variations.

### SOD enzyme activity

The SOD enzyme activity assay result showed that *S. sisymbriifolium* WE extract had the highest enzyme activity at concentrations range between 6.25–1.953 µg/mL (82–50% activity), respectively. However, the EAA and DCM extracts exhibited the lowest enzyme activity of 40 and 23% at the highest concentrations of 125 and 50 µg/mL, respectively. The positive control (ascorbic acid) had significantly higher enzyme activity at concentration ranges of 25–1.563 µg/mL (90–50% activity). The EC_50_ concentrations of the WE, EAA, DCM extracts as well as that of the ascorbic acid were recorded as 68.80; 114.1; 140.2 and 3.0 µg/mL.

### Cytotoxicity effects

**Figure 3:**
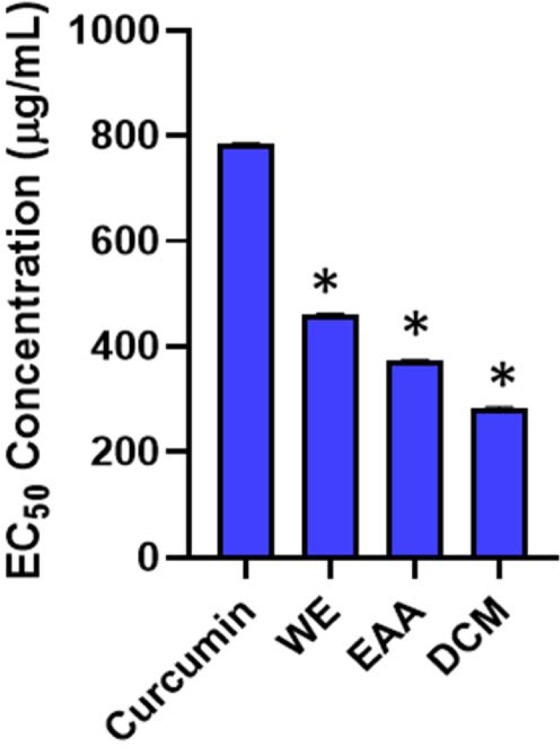
EC_50_ values of *S. sisymbriifolium* leaf extracts on macrophage cells tested at a concentration range of 2.0–250 µg/mL and Curcumin 2.0–250 µM. Cell proliferation assay were performed in triplicate and the mean ± SD were calculated with *p < 0.05. The bar graphs with asterisks (*) denotes a significant difference (p ≤ 0.05) when compared to the control based on Duncan’s multiple comparison test.

The cytotoxic effects of *S. sisymbriifolium* extracts and the positive control (curcumin) were evaluated at concentrations ranging from 2.0–250 µg/mL and 2.0–250 µM, respectively, on macrophage cells line by MTT assay. The extracts and curcumin showed relatively low toxicity with cell viability index >75%. The lethal concentration at 50% (LC_50_), which is the lowest concentration of the extracts that inhibits 50% of the cells, was calculated using the trendline coefficient with the goodness of fit (R^2^) ≥ 0. 900. The calculations indicated that the WE extract exhibited the lowest LC_50_ of 462 µg/mL, followed by the EAA extract (373.4 µg/mL) and DCM extract being the highest (284.5 µg/mL) after 24 h of exposure. The positive control (Curcumin) showed higher LC_50_ of 785 µg/mL (Fig. 3). In theory, the lowest LC_50_ value indicates the highest toxicity and therefore, the extract with the highest LC_50_ is the most preferable when tested on normal immune cells. Bhuyan^25^ reported the toxicity effects of *S. sisymbriifolium* extracts and its fractions on brine shrimp with LC_50_ values recorded for ethanol extract (61.66 µg/mL), hexane (38.90 µg/mL), chloroform (13.97 µg/mL), carbon tetrachloride (203.33 µg/mL), aqueous fractions (247.64 µg/mL) while the positive control, potassium permanganate (KMnO_4_) exhibited an LC_50_ value of 11.90 µg/mL.

### Real-time cell analysis (RTCA)

**Figure 4:**
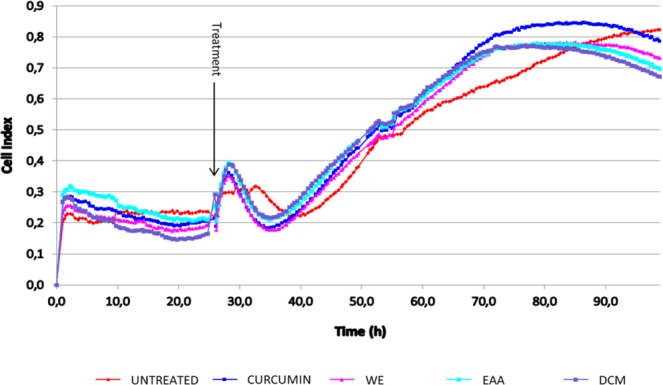
Monitoring the effect of *S. sisymbriifolium* (WE, EAA and DCM) extracts (100 µg/mL) on the viability of macrophage cells during 72 h exposure by the RTCA system.

The xCELLigence RTCA system uses microelectronic biosensors to monitor real-time cellular events such as cell number change, adhesion, viability, morphology and motility^26^. The electrical impedance of the cell population in each well was measured and converted to cell index values (CI), which is the quantitative measure of cell growth exposed to extracts. In our study, the change in CI values during the first 24 h of the experiment can be interpreted as cell adhesion and spreading. Treatment with extracts and curcumin (positive control) was administered after 24 h of incubation. One concentration of each extract (100 µg/mL) which was not toxic to cells when evaluated on MTT assay was subjected to RTCA analysis. Results observed in the RTCA plot (Fig. 4) indicate that extracts (100 µg/mL) continuously increased cell growth, although the cytotoxic patterns of the extracts were observed after 72 h, where the CI value decreased significantly compared to untreated cells. The growth curve of the positive control Curcumin (10 µM) was slightly higher than the extract-treated cells with an approximate CI value of 80% at 72 h.

### H_2_DCF-DA ROS detection

**Figure 5:**
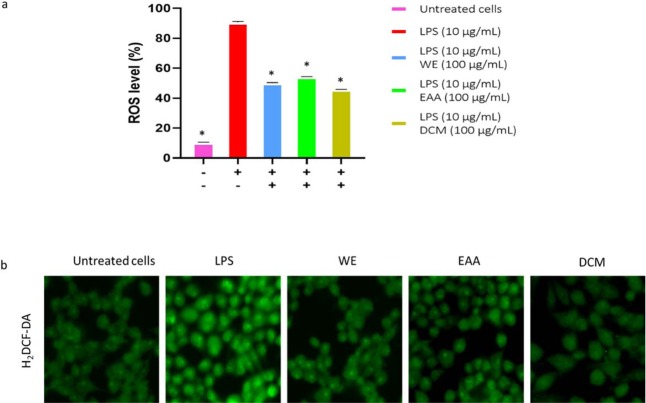
Effective inhibition of LPS-induced ROS production by *S. sisymbriifolium* extracts in macrophage cells **(a)**. Statistical analysis: one-way ANOVA with Duncan multiple range test for means ± SD separation where *P < 0.05 was considered to indicate statistically significant difference compared to the LPS-treated group. **(b)** Images of the effects of LPS-induced ROS production by extracts imaged by confocal microscope using the H_2_DCFDA dye.

The H_2_DCF-DA method was used to determine the LPS-induced ROS production due to oxidative stress in living cells. The quantification of ROS produced was correlated to the fluorescence intensity. The *S. sisymbriifolium* extracts tested at 100 µg/mL significantly reduced the LPS-induced ROS production in Raw 264.7 cells treated with extracts WE, EAA and DCM showing 50, 54 and 45% reduction, respectively. However, the LPS-treated (10 µg/mL) cells showed an increase in ROS production with approximately 90% ROS level compared to non-stimulated cells (Fig. 5a). Reduction of LPS-induced ROS production was further imaged on a confocal microscope (Fig. 5b) where cells were exposed to extracts (100 µg/mL), LPS and untreated cells served as controls. Images revealed a similar trend as those of ROS reduction as in Fig. 5a. This reduction of ROS production signifies the therapeutic effectiveness of *S. sisymbriifolium* as a potential drug for the treatment of neurodegenerative diseases.

### Characterization of phytochemicals by FTIR

**Figure 6:**
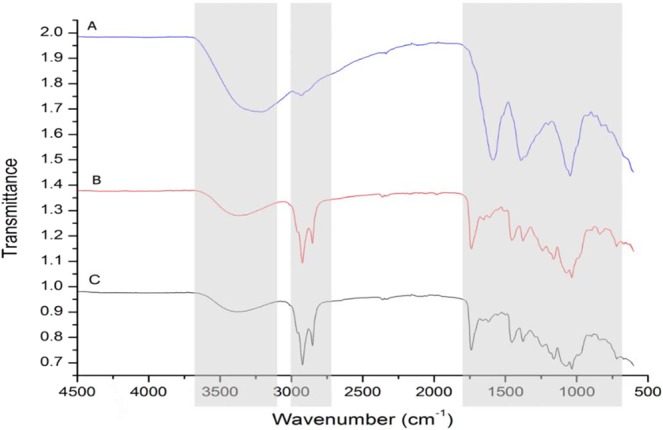
FTIR spectra of 50% water-ethanol **(A)**, ethyl-acetate **(B)** and dichloromethane **(C)** extract of *S. sisymbriifolium* leaves. The shaded portions show the differences between the three extracts.

The FTIR results revealed peak values representing functional groups which were indicated by the wavenumber and percentage of transmittance (Fig. 6). Various functional groups in the extracts are shown by variations in the peaks, which represent metabolites that may significantly contribute to the antioxidant properties in the extracts. The peak at 3250 represents the O–H stretching vibrations of O-H or–NH stretching of amine moieties in WE extract^27^. However, it is worth noting that the peak at 3250 in the WE extract had shifted in the EAA and DCM extracts to 3450 cm^−1^, which may be due to –NH group. The smaller peak at 2920 cm^−1^ demonstrates –CH stretch of the alkanes and – OH stretch of the carboxylic acids. Peaks displayed at 2900 cm^−1^ and 2850 cm^−1^ correspond to the C–H stretching and CH_2_ groups^28^. The strong peaks at 1750 cm^−1^ represent the C=O stretch, which corresponds to the esters or saturated aliphatic type metabolites found in the mid-polar (EAA) and less polar (DCM) extracts. The phenyl rings with a –CH bending type of structure are shown by a strong sharp single peak at 1586 cm^−1^ and aromatic rings at 1388 cm^−1^ ^29,30^. Peaks at 1352 cm^−1^ were depicted as alkanes (-CH_3_) bending and amines at the C-N stretching at 1200 cm^−1^ ^27,28,31^. The EAA (red trace (b) in Fig. 6) and DCM (black trace (c) in Fig. 6) spectra also showed the presence of an acetate ester [-C-C (O)-C] at 1158 cm^−1^. This analysis of metabolites supports previous studies that were conducted on *S. nugrum*^32^ and *S. trilobatum*^33^. The FTIR analysis of the leaf extracts of *S. sisymbriifolium* showed the presence of multiple functional groups such as alcohols, alkynes, alkenes, amines, esters and carboxylic acids, which probably represent different phytochemical classes which include but are not limited to alkaloids, phenols and flavonoids. The compilation of secondary metabolites isolated from *S. sisymbriifolium* WE extract displayed classes of compounds belonging to amines and carboxylic acid groups, which include alkaloids (solacaproine, solanine, solasodine), poly-phenol (neoligan) and steroids (cilistadiol, cilistol)^34,35^ which is consistent with the FTIR results obtained in this study.

## Discussion

This study has demonstrated that *S. sisymbriifolium* extracts exhibit a dose-dependent decrease in inhibition of free radicals and enzyme activity. WE extract showed strong DPPH and ABTS radical scavenging activity and inhibited the SOD enzyme activity at the highest tested concentrations, but at lower concentrations, enzyme activity was more expressed. These results suggest that *S. sisymbriifolium* can be a source of natural antioxidants. The extracts were relatively non-toxic to macrophage cells and the RTCA showed a significant decrease in viable cell index after 70 hrs which suggests toxicity. All three extracts neutralized the LPS-induced ROS production, and this places *S. sisymbriifolium* in a category of potential drug leads for treatment and management of neurodegenerative diseases. The therapeutic potential of *S. sisymbriifolium* can be attributed to the presence of phytochemicals including alcohols, carboxylic acids and amine groups that may be responsible for radical scavenging activity and ROS neutralization. Results presented in this study authenticate the use of *S. sisymbriifolium* to treat various ailments. Our future prospective work aims at investigating the extracts on the activity of other enzymes including catalase glutathione peroxidase and lipase, inhibition of inflammatory cytokines, including TNF-*α*, IL-6, and IL-1*β* which are involved in the progression of oxidative diseases.

## Materials and methods

### Reagents and cell-line

DPPH, ABTS, MTT, LPS, DMSO, Curcumin and SOD kit were products purchased from Merck SA (Pty) Ltd. (Sandton, Johannesburg, SA). Methanol, ethyl acetate dichloromethane and H_2_DCF-DA were purchased from Sigma Aldrich Chemicals Co. (St. Louis, Missouri, USA). All cell culture consumables and reagents were bought from either Promo-Lab Separations (Pty) Ltd (Randburg, South Africa) and RAW 264.7 macrophage cells were obtained from Cellonex (Separation Scientific (Pty) Ltd, Honeydew, South Africa).

### Plant collection and extraction

*Solanum sisymbriifolium* was collected at the University of South Africa, Florida Campus. The plant was deposited at the University of Pretoria Herbarium, where voucher specimens were obtained for identification and authentication. The leaves of the plant were dried at 25 °C and pulverized into a fine powder using an IKA®-MF10.2 grinding mill (Germany - IKA® Werke GmbH & Co. KG). Ten grams of powdered plant material was extracted twice with dichloromethane (DCM), ethyl-acetate (EAA) and 50% ethanol (WE), respectively for 24 hours on a shaker. Thereafter, the extracts were filtered using a Whatman filter paper on a vacuum Buchner funnel, and the filtrates were concentrated using a GeneVac™ EZ-2 concentrator (SP Scientific).

### DPPH scavenging activity assay

The free radical scavenging activity was done using the DPPH scavenging photometric assay. The extracts along with the positive control (ascorbic acid) were reconstituted in methanol to obtain an initial concentration of 1 000 µg/mL and were then mixed with 90 mM of methanolic DPPH to form a final extract and ascorbic acid concentrations of 250; 125; 62.5; 31. 25; 15. 625; 7. 813; 3. 906 and 1. 953 µg/mL in a 96 well plate. The plates were incubated for 30 min at 25 °C and the absorbance was read in a microplate reader (Varioskan Flash, Thermofisher Scientific) at a wavelength of 517 nm. DPPH inhibitory Percent was calculated as following the formula below.

$${\rm{DPPH}}\,{\rm{scavenging}}\,{\rm{percent}}( \% )=[{\rm{control}}\,{\rm{A}}0.-{\rm{sample}}\,{\rm{A}}1./{\rm{control}}\,{\rm{A}}0.\,\,]\times 100$$

where control A0. and sample A1. are absorbances of the DPPH without the extract and DPPH with the extract, respectively.

### ABTS radical scavenging activity

The determination of free radical scavenging activity of plant extracts and ascorbic acid was done by ABTS radical cation decolorization assay. ABTS cation radical production was accomplished by combining 10 mg of ABTS and 2 mg potassium persulfate in water. The solution was kept in the dark at room temperature for approximately, 12–16 h before use. ABTS solution (1 mL) was then diluted with 60 mL of methanol. The experiment was conducted following the method described in the DPPH Scavenging Activity Section. Measurements were then gradually carried out in triplicates. The percentage of inhibition of absorbance at 734 nm was calculated using the formula stated by Prior *et al*.^36^.

$${\rm{ABTS}}\,{\rm{scavenging}}\,{\rm{percent}}( \% )=[{\rm{control}}\,{\rm{A}}0.-{\rm{sample}}\,{\rm{A}}1./{\rm{control}}\,{\rm{A}}0.\,\,]\times 100$$

where control A0. and sample A1. are absorbances of the ABTS without the extract and DPPH with the extract, respectively.

### SOD enzyme activity assays

SOD activity was determined using the SOD assay kit-WST (Sigma-Aldrich®), which is used to measure the rate of inhibition of the SOD enzyme. The reaction mixtures in the SOD kit were combined with 20 μL of working solution sample and thereafter, were gently shaken before being incubated at 37 °C for 20 min. The inhibition activity of SOD on the reaction of xanthine oxide generated the superoxide with a tetrazolium salt which was then determined by measuring the absorbance of the mixtures at a wavelength of 450 nm using a microplate reader. In this study, the positive control was ascorbic acid (10 mg/mL) in place of the extracts was used.

### MTT cytotoxicity assay

The MTT (3- [4, 5-dimethylthiazol-2-yl]-2, 5 diphenyl tetrazolium bromide) cell proliferation assay involves the conversion of MTT into formazan crystals by living cells. The reduction of MTT to formazan by mitochondrial succinate dehydrogenase enzyme activity is the hallmark of measuring cell viability or its death. The assay was conducted following a slightly modified version of the method described by^37^. The macrophage cells (Raw 264.7) were seeded into a 96 well plate at of 1 × 10^4^ cells/well and incubated for 24 h at 37 °C in 5.0% CO_2_ saturation to allow attachment prior to treatment. The cells were treated with various gradients of extract concentrations ranging from 100–3. 125 µg/mL, while curcumin was used as a positive control with a concentration range of 0.2 to 0. 003125 mM. The 96 well plates were incubated for 48 h, after which, MTT (5 mg/mL) was added and the plates were incubated for a further hour. Dimethyl sulfoxide (DMSO) was used as a solubilizing agent to dissolve the formazan and the plates were spectrophotometrically measured at a wavelength of 570 nm using a microplate reader (Varioskan Flash, Thermofisher Scientific). The percentage of cell viability was calculated using the formula below

$${\rm{Cell}}\,{\rm{viability}}( \% )=[{\rm{AT}}/{\rm{AC}}]\times 100$$

where AT and AC represents the absorbance of the extract-treated cell and untreated control cells, respectively.

### Real-time cell analysis (RTCA) of cytotoxicity

The real-time effects of plant extracts on macrophage cells (Raw 264.7) cells was monitored using the RTCA-DP system (xCELLigence, ACEA Biosciences, Roche Applied Science). Briefly, the cells were grown in the cell culture flasks until reaching 80% confluency and then seeded (1 × 10^4^) into the E-plate 96 wells and incubated for 24 h prior to treatment. Cells were treated with extracts at the concentrations of 250 µg/mL, and a positive control (Curcumin) was included at a concentration of 10 µM and untreated cells served as a negative control. This experiment was done in duplicates and run for 72 h.

### H_2_DCF-DA ROS detection assay

The assessment of ROS was done using the macrophage cells (RAW 264.7) and the method followed was by Chen *et al*.^3^ with minor modifications. Cells were seeded at 6 × 10^4^ cells/well in a 96 well plate and incubated for 24 h at 37 °C in 5% CO_2_. After incubation, cells were treated with extract concentration of 100 µg/mL with LPS (10 µg/mL) serving as the positive control. The ROS stimulatory and non-toxic doses of LPS were used^38^. After 24 hrs incubation, 10 µM of H_2_DCF-DA was added for 30 min in the dark. The fluorescence was measured using a microplate reader at 485 and 535 nm excitation and emission, respectively. Similarly, cells cultured on a coverslip in 6 well plates were subjected to similar treatments and the slides were imaged using a confocal laser scanning microscope (LSM 710, Zeiss, Germany).

### Characterization of phytochemicals by FTIR

Functional groups that may have been responsible for the antioxidant activity were determined using the Fourier-Transform Infrared Spectroscopy (FTIR) (Vertex 7, Bruker). Extracts derived from the dried powder or paste were placed into the sample chamber of the FTIR and the spectra were recorded in the range of 4500–500 cm^−1^. Thirty-two scans were performed on each sample with a resolution of 4 cm^−1^. Data were processed using OriginPro 8.1 software (Northampton, Massachusetts, USA) and the results obtained from the graphs were compared with data from previously reported publications.

### Data analysis

Data analysis using GraphPad Prism version 8.2 software (GraphPad Software, CA, USA). One-Way analysis of variance was used to compare means and the results were expressed as the mean ± SD. Differences between means were separated using the Duncan’s multiple range test at P < 0.05. Images were obtained using a Confocal Laser Scanning Microscope (LSM 710; Axio Observer.Z1; Zeiss, Germany) and analyzed with Zen software (Germany).
